# 

**Published:** 1889-08-31

**Authors:** 


SATURDAY, AUGUST 31st, 1889.

**Children and Creeds** - - - - - - - - 335
**Words of Consolation**.—Other Lessons of Sickness - 336
**On Some Points Connected with Cholera**.—Modern Theories - - - - - - - - 337
**The Story of the Insane from Year to Year**.—Asylum Reports - - - - - - - 338
**Turning Over New Leaves**.—Daphne's Daring - - 338
**The Doctor's Pulpit**.—In Nature's School - - - - 339
**The Boarding Out of Pauper Children**. I.—The System Itself - - - - - - - - 340
**Within the Wards**.—Curiosities of Brain and Nerve - 341
**Notes and News** - - - - - - - - - 342
**Everybody's Page** - - - - - - - - 344
**Annotations**.—Doctors and Paupers—Disinfectants—From Hand to Mouth - - - - - - - - 345
**The Plans and Purposes of the Johns Hopkins Hospital**. I. — A Training School for Nurses. By JOHN S. BILLINGS, M.D., Surgeon, U.S. Army - - - 346
**The Hospital Workshop**. VIII.—Out-Patients at the Brompton Hospital - - - - - - - - - 347
**Her Heart's Mistake**. By E. D. CUMMING. Chap. xx. - 348
**Amusements—Scraps and Gleanings** - - - - 350

**EXTRA SUPPLEMENT—THE NURSING MIRROR.**

**En Passant**.—Short Items—Home Nursing—Asylum Attendants—A Nursing Guild—Homes Wanted—Doll Competition—To Members of the B. N. A.—Summer Afternoons - lxxxiii
**How to become a Nurse** - - - - - - lxxxiv
**Everybody's Opinion**.—Pensions—Examination Questions—Clean Nails - - - - - - - - lxxxv
**Physiology for Nurses**. No. 7.—Spinal Complaints - lxxxv
**Winter Amusements and Remunerative Work**.—An Exhibition of Doll Nurses - - - - - lxxxvi
**Appointments—Presentation** - - - - - lxxxvi
**Death in Our Ranks** - - - - - - - lxxxvi
**Vacancies—Notes and Queries** - - - - lxxxvi

